# Advances in the application of wearable sensors for gait analysis after total knee arthroplasty: a systematic review

**DOI:** 10.1186/s42836-023-00204-4

**Published:** 2023-10-02

**Authors:** Yuguo Feng, Yu Liu, Yuan Fang, Jin Chang, Fei Deng, Jin Liu, Yan Xiong

**Affiliations:** 1 College of Art and Design, Xihua University, Chengdu, 610039 China; 2 Chongqing Brace Technology Co., Ltd., Chongqing, 401120 China; 3 Affiliated Experimental School of Sichuan Normal University, Chengdu, 610000 China; 4 Department of Orthopaedics, Daping Hospital, Army Medical University, Chongqing, 400042 China

**Keywords:** Wearable sensors, Total knee arthroplasty, Walking, Gait analysis, Review

## Abstract

**Background:**

Wearable sensors have become a complementary means for evaluation of body function and gait in lower limb osteoarthritis. This study aimed to review the applications of wearable sensors for gait analysis after total knee arthroplasty (TKA).

**Methods:**

Five databases, including Web of Science Core Collection, Embase, Cochrane, Medline, and PubMed, were searched for articles published between January 2010 and March 2023, using predetermined search terms that focused on wearable sensors, TKA, and gait analysis as broad areas of interest.

**Results:**

A total of 25 articles were identified, involving 823 TKA patients. Methodologies varied widely across the articles, with inconsistencies found in reported patient characteristics, sensor data and experimental protocols. Patient-reported outcome measures (PROMs) and gait variables showed various recovery times from 1 week postoperatively to 5 years postoperatively. Gait analysis using wearable sensors and PROMs showed differences in controlled environments, daily life, and when comparing different surgeries.

**Conclusion:**

Wearable sensors offered the potential to remotely monitor the gait function post-TKA in both controlled environments and patients’ daily life, and covered more aspects than PROMs. More cohort longitudinal studies are warranted to further confirm the benefits of this remote technology in clinical practice.

## Background

Knee osteoarthritis (KOA) is a common degenerative joint disease affecting articular cartilage, menisci, capsule, and other soft tissues [1]. It significantly reduces the quality of life in approximately 10% of KOA patients aged over 60 years [2]. Total knee arthroplasty (TKA) is the most effective treatment for severe knee joint diseases, but selecting an appropriate tool for the assessment of postoperative outcomes is challenging [3].

The patient-reported outcome measures (PROMs) are the commonly used questionnaires to assess postoperative knee pain, keen function, patient satisfaction, etc. However, PROMs should be used in randomized controlled trials due to inherent ceiling effects, poor patient-clinician communication, and unrepeatable results [2]. Optical gait analysis is an objective and quantitative tool to provide detailed kinematic measurements, but the complexity, cost and inconvenience associated with the method impede its widespread application in clinical practice [4]. Wearable sensors are miniaturized and low-cost monitoring devices for real-time detection of movements and posture. Biomechanical data can be collected from accelerometers, gyroscopes, and magnetometers attached to different parts of the body [5]. Therefore, gait analysis, in combination with the use of wearable sensors provides a convenient, efficient, and inexpensive means for data collection, allowing for high-accuracy gait feature extraction for analysis [6]. To our knowledge, only a few systematic reviews [7, 8] have pooled the available evidence for the assessment of post-TKA rehabilitation by combining the technologies, but they have not focused on TKA or gait analysis.

This systematic review aimed to present more data about the application of gait analysis in combination with wearable sensor technologies in post-TKA rehabilitation.

## Materials and methods

### Inclusion and exclusion criteria

The scoping review was conducted in accordance with the preferred reporting items of the meta-analysis (PRISMA) guidelines [9]. Articles that satisfied all of the following criteria were included in this study: (1) gait analyses with wearable sensors; (2) post-TKA management; and (3) studies published between January 2010 and March 2023. Articles that met one of the following criteria were excluded (1) conference abstracts; (2) review articles; (3) non-TKA treatment; (4) the lack of gait or biomechanical data; (5) studies on technological evaluation; (6) the lack of wearable sensors; (7) robot-assisted rehabilitation or the use of a surgical navigation system; (8) non-independent walking; and (9) the absence of full text.

### Search and selection strategies

A systematic search was conducted in the Web of Science Core Collection, Embase, Cochrane, Medline and PubMed. The pre-determined search terms for this review were: wearable electronic devices, total knee arthroplasty and gait analysis. Search strategies for each database were detailed in Appendix. In addition, other relevant articles were also searched in order to find relevant references.

Upon comprehensive searching, duplicate articles were automatically removed using the Endnote software, and the duplicates were verified by the first author (Y.G.F.). Using the software, two authors (Y.G.F. and Y.L.) selected the articles by reviewing the titles and abstracts. The articles were finally confirmed on the basis of review of the main text. The first author (Y.G.F.) collected the data from the main text of the articles. The data were validated by the second author (Y.L.). Disagreements were resolved by comparing notes and reaching a consensus between the two authors (Y.G.F. and Y.L.) and the third author (Y.X.).

## Results

### Selected articles

**Fig. 1:**
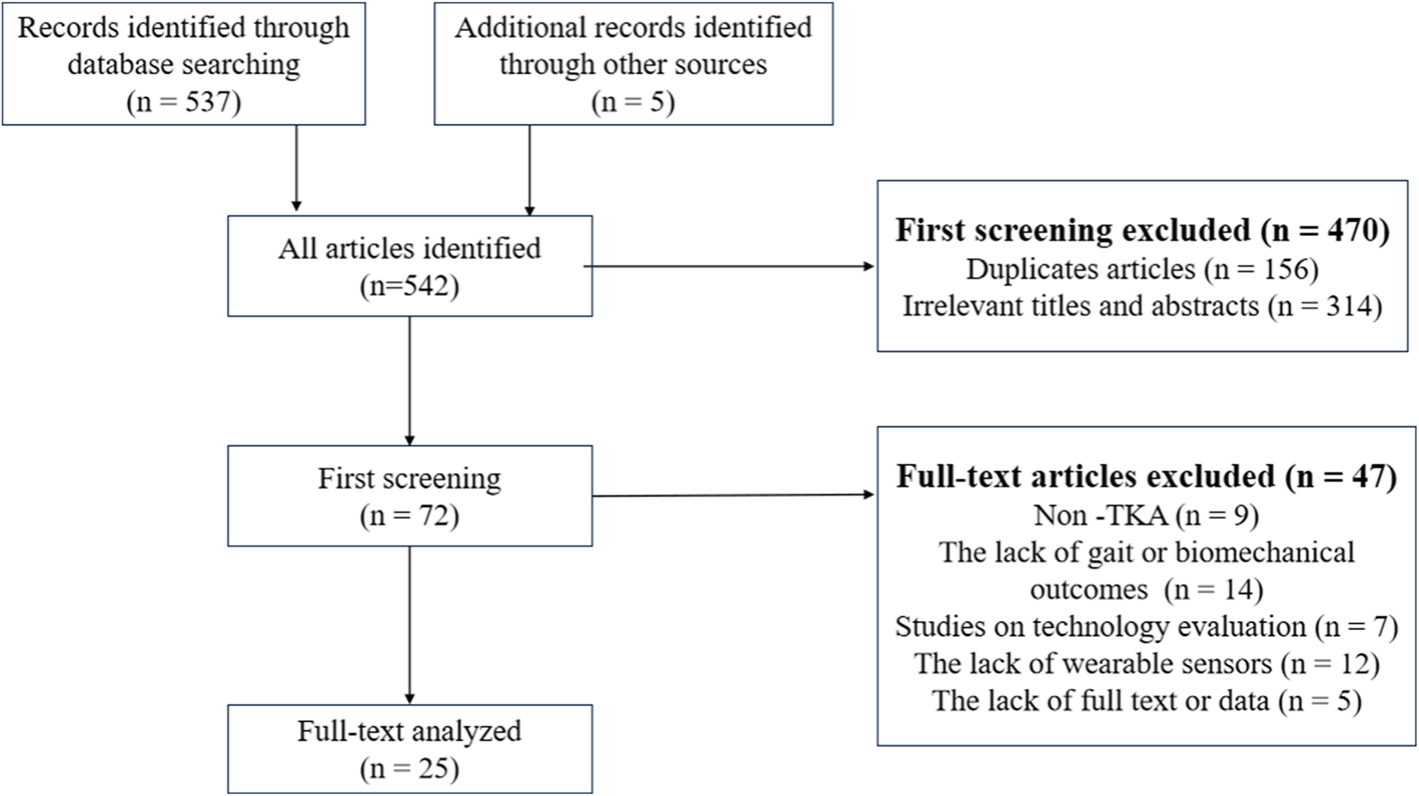
PRISMA flow diagram

We identified 542 articles (537 articles from the databases and 5 articles from other sources) by using the aforementioned search strategy. We removed 156 duplicate articles. We excluded 361 articles according to the exclusion criteria. Finally, 25 articles were included in the study. The PRISMA flowchart is shown in Fig. 1.

### Patient characteristics

We finally identified 25 articles (823 patients), including unilateral [10–21], bilateral [14], cruciate-retaining [4] and posterior-stabilized [4, 18], bi-condylar [22], bi-cruciate retaining and bi-cruciate stabilized [23, 24], fixed-bearing prostheses and mobile-bearing prostheses [25], medial-pivot [18], posterior stabilized [24], minimally invasive [26], revision [17] and primary TKA [11, 16, 17, 27, 28], as well as “unknown techniques” [29–31] (Table 2).

### Sensors

Sensory data varied widely (Table 1). Sampling frequencies ranged from 32 Hz to 1,149 Hz. Frequencies were less than 100 Hz in 2 articles [11, 30]. No frequency was reported in 7 studies [21, 23–26, 28, 31]. The most common location of sensors was the torso (*n* = 17) [4, 11, 15, 18–30, 32], followed by the foot (*n* = 7) [12, 15, 17, 21, 22, 24, 29], thigh (*n* = 7), lower leg (*n* = 6) [13, 16, 22, 25, 31, 33] and head (*n* = 1) [18].

**Table 1:** Summary of the samples and protocols of the 25 articles included

**Authors**	**Sensors**			**Study Design**			**Gait variables**					
	**Total no of sensors**	**Frequency (Hz)**	**Body location**	**Walking distance (Meters)**	**6 MWT**	**Experimental environment**	**Spatiotemporal parameters**	**Range of motion of the knee**	**Acceleration**	**Step time asymmetry**	**Coefficient of variation**	**Kinetic parameters**
Amemiya [23]	1	/	B	10		I	**√**				**√**	
Boekesteijn [29]	4	128	B/F	6		I	**√**	**√**	**√**	**√**	**√**	
Bolam [10]	2	1149	F	/	**√**	O		**√**				**√**
Bolink [4]	1	100	B	20		I	**√**			**√**	**√**	
Brandes [11]	3	32	B/T	/		O	**√**					
Calliess [22]	3	100	B/T/L	100		I	**√**	**√**			**√**	
Çankaya [12]	2	1149	F	25		I	**√**			**√**		
Chapman [33]	2	128	T/L	/		O/I	**√**	**√**				
Christiansen [13]	2	1000	L	/	**√**	I			**√**			
Daugaard [14]	1	100	T	/		O	**√**					
Emmerzaal [30]	1	60	B	10		I	**√**		**√**			
Fransen [27]	1	100	B	100		I	**√**			**√**	**√**	
Fransen [32]	1	100	B	/		O	**√**		**√**			
Hiyama [16]	1	500	L	16		I	**√**	**√**			**√**	
Hiyama [15]	2	500	B/F	16		I	**√**		**√**			
Jolles [25]	5	/	B/T/L	30		I	**√**	**√**				
Kluge [17]	2	102	F	40		I	**√**					
Lee [28]	1	/	B	8	**√**	I	**√**	**√**	**√**			
Lo [18]	2	128	B/H	20		I	**√**		**√**	**√**		
Rahman [31]	2	/	T/L	20		I	**√**	**√**				
Senden [19]	1	100	B	20		I	**√**			**√**		
Storey [20]	1	100	B	10	**√**	O/I	**√**					
Tomite [24]	2	/	B/F	50		I	**√**	**√**				
Tsuji [26]	1	/	B	10		I			**√**			
Zhang [21]	4	/	B/T/F	40		I	**√**					

*B* Torso, *F* Foot, *I* Indoors, *L* Lower leg, *O* Outdoors, *T* Thigh, *6-MWT* 6-min walk test

### Study designs

Walking was different in 25 articles. Non-walking protocols were reported in 4 articles [11, 14, 32, 33]. Walking distances ranged from 10 to 100 m in 19 articles. 6-min walk time was reported in 4 articles [10, 13, 20, 28]. Furthermore, the most common experimental environment was indoors (*n* = 19), followed by outdoors (*n* = 4) [10, 11, 14, 32], and then by both indoors and outdoors (*n* = 2) [17, 20]. The study design is detailed in Table 1.

### PROMs and gait outcomes

More than one PROMs were employed in most of the 25 articles. The most common PROMs were EuroQol Five-Dimensions Questionnaire (EQ-5D), Knee Injury and Osteoarthritis Outcomes Score (KOS), Knee Society Score (KSS), Oxford Knee Score (OKS), Visual Analogue Scale (VAS), and Western Ontario and McMaster Universities Osteoarthritis Index (WOMAC) (Table 2).

**Table 2:** Summary of follow-up time of the 25 articles

Authors	**Populations**	**Time points**	**PROMs**	**Gait outcomes**
**Conventional studies**				
Boekesteijn [29]	TKA (*n* = 24)	2 months 15 months	KOOS: continuous improvement	Improved after 2 months
Bolink [4]	Cruciate-retaining TKA (*n* = 13); Posterior-stabilized TKA (*n* = 7)	12 months	WOMAC/KSS: improved	Improved
Christiansen [13]	Unilateral TKA (*n* = 24)	5 weeks 26 weeks	/	Reached the normal levels at 26 weeks
Calliess [22]	Bi-condylar TKA (*n* = 4)	12 months	KSS/OKS: improved	Improved
Emmerzaal [30]	TKA (*n* = 21)	6 weeks 3 months 6 months 12 months	KOOS: did not reach the normal level at 12 months	Reached the normal level at 6 months
Fransen [27]	Primary TKA (*n* = 65)	12 months	OKS: improved	Improved
Hiyama [16]	Primary unilateral TKA (*n* = 57)	1 week	Pain assessment: significantly decreased	No improvement in stride time variability
Hiyama [15]	Unilateral TKA (*n* = 27)	6 months	/	Decreased
Kluge [17]	Unilateral TKA (*n* = 24)	12 months	WOMAC/OKS/KSS/EQ-5D/WHO Disability Assessment Schedule 2.0 scores: improved	No significant change
Lee [28]	Primary unilateral TKA (*n* = 84)	1 month	WOMAC/EQ-5D/VAS: improved	Improved
Rahman [31]	TKA (*n* = 27)	2 months 12 months	OKS: improved at 12 months	No significant improvement at 12 months
Senden [19]	Unilateral TKA (*n* = 12)	2 weeks 6 weeks 3 months	WOMAC/KSS/VAS/Pain Disability Index: improved	Improved at 3 months
Zhang [21]	Unilateral TKA (*n* = 12)	6 weeks 6 months	American KSS: significant improvement	No significant change
**Surgical comparison studies**				
Amemiya [23]	Bi-cruciate retaining TKA (*n* = 10); Bi-cruciate stabilized TKA (*n* = 10)	6 weeks 3 months	/	Significant difference at 6 weeks; No significant difference at 3 months
Çankaya [12]	Unilateral TKA (*n* = 34)	12 months	/	Significantly difference
Jolles [25]	Fixed-bearing prostheses TKA (*n* = 29); Mobile-bearing prostheses TKA (*n* = 26)	6 weeks 3 months 6 months 12 months 5 years	WOMAC/KSS/EQ-5D/VAS: significant difference	Age was a major factor in the difference between two prostheses
Lo [18]	Medial-pivot TKA (18) posterior-stabilized TKA (20)	12 months	WOMAC: no significant difference	Significantly difference in anteroposterior sway of the lumbar and head regions
Tomite [24]	Bi-cruciate stabilized TKA (*n* = 30); Posterior stabilized TKA (*n* = 30)	12 months	New KSS: significant difference	Significantly difference
Tsuji [26]	Minimally invasive surgery TKA (*n* = 10); Standard TKA (*n* = 10)	1–4 weeks	VAS: no significant difference	Significant difference in cumulative acceleration
**Daily life studies**				
Bolam [10]	Unilateral TKA (*n* = 14)	2–6 weeks	OKS/EQ-5D/VAS: significant improvement	Improve at 6 weeks
Brandes [11]	Primary unilateral TKA (*n* = 53)	2 months 6 months 12 months	KSS/Short Form-36 Health Survey: improved	No significant improvement after 6 months
Chapman [33]	Revision TKA (*n* = 2); Primary TKA (*n* = 8)	1–6 weeks	Mental/Physical Component Scores/KOOS/Pain: improved	Significant differences between indoors and outdoors
Daugaard [14]	Unilateral TKA (*n* = 40); Bilateral TKA (*n* = 12)	5 years	KOOS: improved	No improvement in daily short walking bouts
Fransen [32]	Unilateral TKA (*n* = 38)	3 months	OKS/Modified Gait Efficacy Scale: improved	No significant change
Storey [20]	Unilateral TKA (*n* = 28)	/	Activities of Daily Living: no significant difference	No significant difference

*EQ-5D* EuroQol Five-Dimensions Questionnaire, *KOOS* Knee Injury and Osteoarthritis Outcomes Score, *KSS* Knee Society Score, *OKS* Oxford Knee Score, *VAS* Visual Analogue Scale, *WOMAC* Western Ontario and McMaster Universities Osteoarthritis Index

Gait parameters included the length, width, speed, and frequency of stride. Other parameters included the range of motion (ROM) of the knee (*n* = 9) [10, 16, 22, 24, 25, 28, 29, 31, 33], acceleration (*n* = 8) [13, 15, 18, 26, 28–30, 32], the step time symmetry (*n* = 6) [4, 12, 18, 19, 27, 29], coefficient of variability (*n* = 6) [4, 16, 22, 23, 27, 29] and kinetic parameters (*n* = 1) [10] (Table 1).

In 13 studies, PROMs improved from the second postoperative week [19] to the 12th postoperative month [4, 17, 22, 27, 29, 31], but gait analysis showed that improvement varied with the different follow-up time points. Storey et al. [28] reported improvement beginning at the 1st-month post-surgery, while Senden et al. [29] observed the change from the 2nd month postoperatively, and yet Tsuji et al. [19] started their observation from the 3rd month. Besides, Çankaya et al. [30] and Tomite et al. [13] pointed out that the patient’s gait returned to normal level 6 months after operation but the notion was not supported by some articles [15, 17, 21]. In most studies, gait parameters improved at 12 months after surgery [4, 22, 27, 29, 30], but Kluge et al. [17] and Rahman et al. [31] didn’t agree with the findings (Table 2).

In 6 comparison articles [12, 18, 23–26], wearable sensors generated different gait parameters in different groups, and 2 of the articles reported PROMs [24, 25].

Improved PROMs were found at all follow-up time points. In 6 articles [10, 11, 14, 20, 32, 33], the findings of remote gait assessment differed but all sensors captured the changes in gait parameters at all follow-up time points (Table 2).

### Time points

In the articles, gait was evaluated from 1 week to 5 years postoperatively, with the most common follow-up time points being 6 weeks, 3 months, 6 months and 1 year after surgery. However, in few studies, follow-up lasted for more than 1 year postoperatively (Table 2).

## Discussion

In this review study, we extracted post-TKA data about patient features, sensor data, study protocols, PROMs, and gait variables at various follow-up time points. However, so far, no standard testing method is available for the assessment of wearable sensor-based gait. Kinematic parameters are the most common gait variables. The post-TKA PROMs showed continuous improvement from 1 week to 5 years, but wearable sensors-based gait outcomes varied substantially with different testing protocols used and other relevant factors [34]. In addition, surgeons can track a wide range of daily gait parameters using remote wearable sensors. These parameters are more sensitive and objective than PROMs. Improvement in PROMs is not consistent across the gait parameters due to the lack of high correlation [4, 16, 19, 21, 27]. In addition, wearable sensor assessments show different TKA techniques produce different gait parameters, and some are not covered by PROMs [12, 23, 26, 27]. Those findings suggested that the functional assessment using PROMs may not accurately reflect a patient’s true mobility, and wearable sensor-based gait assessment can serve as a supplement to make up for the PROM insufficiency [35].

As the most important biomechanical assessment, kinematic analysis is effective in interpreting and predicting the recovery of postoperative movement [36–38]. However, we found that different post-TKA kinematic parameters were used in the follow-up periods. We found many articles reporting the accuracy, consistency, and responsiveness of wearable sensor-based gait analysis, but they mainly focused on the evaluation of healthy gait based on inconsistent protocols [39–42]. Item-Glatthorn et al. [43] took issue with the use of certain gait parameters (such as walking speed and stride length) since the sensors, test methods, and parameter definitions restricted the comparability of the findings [7]. Hafer et al. [44] also expressed concerns about those inconsistent and unreliable protocols. Similarly, Kobsar et al. [7] suggested that the reliability of wearable devices be verified. Therefore, it is imperative to establish a standardized and generally-accepted testing protocol to yield reliable and comparable results.

As important gait indicators, kinetic parameters are used in gait training and surgical planning [37, 45, 46]. Surprisingly, no article reported the use of wearable system-based kinematic parameters for the assessment of post-TKA gait. Emery et al. emphasized avoiding this limitation and making wearable systems more accessible in clinical practice [1]. Currently, many studies examined the feasibility of dynamic assessments using kinematic parameters. Youn et al. [47] extracted 11 inertial gait variables from accelerometers and successfully predicted four kinetic gait variables (maximum knee flexion moment, maximum knee inversion moment, vertical ground reaction force, and maximum ground reaction force). Konrath et al. [48] proposed a musculoskeletal model based on the data derived from wearable sensors. They assessed knee movement in older adults during activities of daily living. They found that the accuracy of internal knee moments measured using wearable sensors was comparable to that of optical motion capture. He et al. developed a wearable sensory training system and successfully predicted the changes in knee internal joint moments during a walking test in elderly KOA patients [49]. In summary, the relevant articles confirmed that joint torque and related load estimation methods were valid on the basis of wearable sensors, and they provided a novel approach for assessing dynamic parameters and led to improved gait training and surgical planning.

The “white coat effect” was observed in rehabilitation assessments, where gait movements conducted in the presence of surgeons or researchers differed from those conducted in their absence [17]. Emmerzaal et al. [30] reported gait differences between the clinical settings and daily environments. PROMs may not represent daily gait behaviors. Wearable sensors have the advantage of allowing physicians to perform remote unsupervised assessments, both in and out of the clinic, throughout the rehabilitation process [50, 51]. Our results suggest that remote measurement using wearable sensors is more informative than PROMs in terms of a patient’s daily gait. It reduces the number of patients’ clinical visits and optimizes rehabilitation training [52]. In order to assess daily gait function better, Chapman et al. [33] suggested that post-TKA rehabilitation and follow-up periods should be longer than 1 to 2 years [32].

Our review has limitations. First, the small patient groups in some articles and different testing methods compromised the power of the evidence in assessing walking ability after TKA. In the future, wearable sensors may be used to monitor real-life physical activities and gait outcomes in TKA. Second, PROMs and gait parameters are subject to some limitations, which affect measurement accuracy.

## Conclusion

This systematic review confirmed that wearable sensors can be used to monitor post-TKA gait function in unsupervised mode and on remote basis, providing additional clinical measurement methods and diagnostic approaches. More longitudinal cohort studies using wearable sensors could help further improve the assessment of gait function and post-TKA rehabilitation.

## Data Availability

The dataset analyzed in this study is available from the corresponding author on reasonable request.

## Appendix

Complete search strategy

Search strategy individually optimized for each database based on the three broad topics of total knee arthroplasty, wearable electronic devices, and gait analysis joined using the "AND" search command/function.

### PubMed

**Total knee arthroplasty:** ("Arthroplasty, Replacement, Knee"[Majr] OR Arthroplasties, Knee Replacement[Title/Abstract] OR Arthroplasties, Replacement, Knee[Title/Abstract] OR Arthroplasty, Knee Replacement[Title/Abstract] OR Arthroplasty, Replacement, Partial Knee[Title/Abstract] OR Knee Arthroplasty[Title/Abstract] OR Knee Arthroplasty, Total[Title/Abstract] OR Knee Replacement Arthroplasties[Title/Abstract] OR Knee Replacement Arthroplasty[Title/Abstract] OR Knee Replacement, Total[Title/Abstract] OR Partial Knee Arthroplasty[Title/Abstract] OR Partial Knee Replacement[Title/Abstract] OR Replacement Arthroplasties, Knee[Title/Abstract] OR Replacement Arthroplasty, Knee[Title/Abstract] OR Replacement, Total Knee[Title/Abstract] OR Total Knee Replacement[Title/Abstract] OR Unicompartmental Knee Arthroplasty[Title/Abstract] OR Unicompartmental Knee Replacement[Title/Abstract] OR Unicondylar Knee Arthroplasty[Title/Abstract] OR Unicondylar Knee Replacement[Title/Abstract]**).**

**Wearable electronic devices:** ("Wearable Electronic Devices"[Majr] OR Electronic Skin[Title/Abstract] OR Wearable Devices[Title/Abstract] OR Wearable Technology[Title/Abstract]) OR wearable sensor*[Title/Abstract] OR wearable technology[Title/Abstract] OR motion sensor*[Title/Abstract] OR inertial sensor*[Title/Abstract] OR inertial motion capture[Title/Abstract] OR inertial measurement unit*[Title/Abstract] OR body sensor network*[Title/Abstract] OR body worn sensor*[Title/Abstract] OR sensor fusion[Title/Abstract] OR IMU[Title/Abstract] OR MEMS*[Title/Abstract] OR acceleromet*[Title/Abstract] OR gyroscop*[Title/Abstract] OR magnetomet*[Title/Abstract] OR Device, Wearable Electronic[Title/Abstract] OR Electronic Device, Wearable[Title/Abstract] OR Wearable Electronic Device[Title/Abstract] OR Technology, Wearable[Title/Abstract] OR Wearable Devices[Title/Abstract] OR Device, Wearable[Title/Abstract] OR Electronic Skin[Title/Abstract] OR Skin, Electronic[Title/Abstract]).

**Gait analysis:** ("Gait Analysis" [Majr] OR Analysis, Gait [Title/Abstract] OR Gait Analyses [Title/Abstract] OR biomechanic*[Title/Abstract] OR walk*[Title/Abstract] OR spatiotemporal [Title/Abstract] OR kinematic*[Title/Abstract] OR acceleration*[Title/Abstract]).

### Embase

**Total knee arthroplasty:** ('total knee arthroplasty'/exp OR 'arthroplasty, replacement, knee' OR 'arthroplasties, knee replacement':ab,ti OR 'arthroplasties, replacement, knee':ab,ti OR 'arthroplasty, knee replacement':ab,ti OR 'arthroplasty, replacement, partial knee':ab,ti OR 'knee arthroplasty':ab,ti OR 'knee arthroplasty, total':ab,ti OR 'knee replacement arthroplasties':ab,ti OR 'knee replacement arthroplasty':ab,ti OR 'knee replacement, total':ab,ti OR 'partial knee arthroplasty':ab,ti OR 'partial knee replacement':ab,ti OR 'replacement arthroplasties, knee':ab,ti OR 'replacement arthroplasty, knee':ab,ti OR 'replacement, total knee':ab,ti OR 'total knee replacement':ab,ti OR 'unicompartmental knee arthroplasty':ab,ti OR 'unicompartmental knee replacement':ab,ti OR 'unicondylar knee arthroplasty':ab,ti OR 'unicondylar knee replacement':ab,ti).

**Wearable electronic devices:** ('wearable computer'/exp OR 'Wearable Electronic Devices' OR 'Electronic Skin':ab,ti OR 'Wearable Devices':ab,ti OR 'Wearable Technology':ab,ti OR 'wearable sensor*':ab,ti OR 'wearable technology':ab,ti OR 'motion sensor*':ab,ti OR 'inertial sensor*':ab,ti OR 'inertial motion capture':ab,ti OR 'inertial measurement unit*':ab,ti OR 'body sensor network*':ab,ti OR 'body worn sensor*':ab,ti OR 'sensor fusion':ab,ti OR 'IMU':ab,ti OR 'MEMS*':ab,ti OR 'acceleromet*':ab,ti OR 'gyroscop*':ab,ti OR 'magnetomet*':ab,ti OR 'Device, Wearable Electronic':ab,ti OR 'Electronic Device, Wearable':ab,ti OR 'Wearable Electronic Device':ab,ti OR 'Technology, Wearable':ab,ti OR 'Wearable Devices':ab,ti OR 'Device, Wearable':ab,ti OR 'Electronic Skin':ab,ti OR 'Skin, Electronic':ab,ti).

**Gait analysis:** ('gait’/exp OR 'Gait Analysis' OR 'Analysis, Gait':ab,ti OR 'Gait Analyses':ab,ti OR 'biomechanic*':ab,ti OR 'walk*':ab,ti OR 'spatiotemporal':ab,ti OR 'kinematic*':ab,ti OR 'acceleration*':ab,ti).

### Cochrane

**Total knee arthroplasty:** ((Arthroplasties, Knee Replacement):ti,ab,kw OR (Arthroplasties, Replacement, Knee):ti,ab,kw OR (Arthroplasty, Knee Replacement):ti,ab,kw OR (Arthroplasty, Replacement, Partial Knee):ti,ab,kw OR (Knee Arthroplasty):ti,ab,kw OR (Knee Arthroplasty, Total):ti,ab,kw OR (Knee Replacement Arthroplasties):ti,ab,kw OR (Knee Replacement Arthroplasty):ti,ab,kw OR (Knee Replacement, Total):ti,ab,kw OR (Partial Knee Arthroplasty):ti,ab,kw OR (Partial Knee Replacement):ti,ab,kw OR (Replacement Arthroplasties, Knee):ti,ab,kw OR (Replacement Arthroplasty, Knee):ti,ab,kw OR (Replacement, Total Knee):ti,ab,kw OR (Total Knee Replacement):ti,ab,kw OR (Unicompartmental Knee Arthroplasty):ti,ab,kw OR (Unicompartmental Knee Replacement):ti,ab,kw OR (Unicondylar Knee Arthroplasty):ti,ab,kw OR (Unicondylar Knee Replacement):ti,ab,kw)).

**Wearable electronic devices:** ((Electronic Skin):ti,ab,kw OR (Wearable Devices):ti,ab,kw OR (Wearable Technology):ti,ab,kw OR (wearable sensor*):ti,ab,kw OR (wearable technology):ti,ab,kw OR (motion sensor*):ti,ab,kw OR (inertial sensor*):ti,ab,kw OR (inertial motion capture):ti,ab,kw OR (inertial measurement unit*):ti,ab,kw OR (body sensor network*):ti,ab,kw OR (body worn sensor*):ti,ab,kw OR (sensor fusion):ti,ab,kw OR (IMU):ti,ab,kw OR (MEMS*):ti,ab,kw OR (acceleromet*):ti,ab,kw OR (gyroscop*):ti,ab,kw OR (magnetomet*):ti,ab,kw OR (Device, Wearable Electronic):ti,ab,kw OR (Electronic Device, Wearable):ti,ab,kw OR (Wearable Electronic Device):ti,ab,kw OR (Technology, Wearable):ti,ab,kw OR (Wearable Devices):ti,ab,kw OR (Device, Wearable):ti,ab,kw OR (Electronic Skin):ti,ab,kw OR (Skin, Electronic):ti,ab,kw)).

**Gait analysis:** ((Analysis, Gait): ti,ab,kw OR (Gait Analyses):ti,ab,kw OR (biomechanic*):ti,ab,kw OR (walk*):ti,ab,kw OR (spatiotemporal):ti,ab,kw OR (kinematic*):ti,ab,kw OR (acceleration*):ti,ab,kw)).

### Web of Science Core Collection/Medline

**Total knee arthroplasty:** TS = (total knee arthroplasty OR arthroplasty, replacement, knee OR arthroplasties, knee replacement OR arthroplasties, replacement, knee OR arthroplasty, knee replacement OR arthroplasty, replacement, partial knee OR knee arthroplasty OR knee arthroplasty, total OR knee replacement arthroplasties OR knee replacement arthroplasty OR knee replacement, total OR partial knee arthroplasty OR partial knee replacement OR replacement arthroplasties, knee OR replacement arthroplasty, knee OR replacement, total knee OR total knee replacement OR unicompartmental knee arthroplasty OR unicompartmental knee replacement OR unicondylar knee arthroplasty OR unicondylar knee replacement).

**Wearable electronic devices:** TS = (Wearable Electronic Devices OR wearable sensor* OR wearable technology OR motion sensor* OR inertial sensor* OR inertial motion capture OR inertial measurement unit* OR body sensor network* OR body worn sensor* OR sensor fusion OR IMU OR MEMS* OR acceleromet* OR gyroscop* OR magnetomet*).

**Gait analysis:** TS = (gait OR analysis, gait OR gait Analyses or biomechanic* OR walk* OR spatiotemporal OR kinematic OR acceleration).
